# Hugh M. Reid

**Published:** 1911-07-15

**Authors:** 


Hugh M. Reid.—Dr. Reid was born in 1836, and died March 3rd, 1911, at Eatontown, N. J. In 1857 he began the practice of dentistry in Green Castle, Indiana, and removed to Cedarville, Ohio in 1860, where he practiced eight or ten years and then moved to Sandusky, Ohio, where he practiced three or four years. He then moved to Cincinnati where he practiced for eleven years; during five years of this time he was Clinical Professor of Operative Dentistry in the Ohio College of Dental Surgery. In 1881 he moved to Minneapolis and practiced until 1896, when he quit practice and taught prosthetic dentistry in the Dental Department of the University of Minnesota until June 1908, when he moved to Eatontown, N. J., where he bought a farm and where he lived at the time of his death. Dr. Reid was not a scientific or literary student, but in his time he was considered one of the best technical practitioners. He was exceedingly fond of mechanics and in the day of large gold contour restorations he had few equals. He had a most excellent idea of artistic proportions and the skill to produce his ideals. Being a man of strong physical proportions and with such height artistic ideals, he used his great skill to make such gold fillings and restorations as no one to-day would think of undertaking. He was also very fond of prosthetic work and his gold plate work was the envy of all practit oners when he was at his best. He was always interested in dental society work and was always ready to do his part in the way of discussion, clinics, etc. He had a keen and convincing method of argument and was strong in debate on all technical procedures. He was personally a most kind and indulgent father and friend to any one who need a friend or helper. His family consisted of his wife, who died three or four years ago, and two daughters and two sons, One of his sons, Edward, is practicing dentistry in Minneapolis, and his youngest son, Hal Reid, has made quite a name for himself as an actor and writer of plays. The two daughters are married and living in Minneapolis.

Dr. Reid was a man of generous impulses and by his kindly interest and large sympathies he made many and lasting friends, who all remember him with sincere regard. We extract the following from a memorial printed in the June *Review* written by his confreres in the University of Minnesota, which expresses the sentiments of his student in the Ohio College as well:

"Dr. Reid was a man of large physique, full of energy and crowned with a countenance beaming with kindness and good will, which always provoked noble inspirations to those with whom he came in contact. He will long be remembered by the students of the college, who loved to call him "Daddy". His kind and helpful interest in all the boys his ability as an instructor and his strong personality endeared him to his students, and when they came to re-visit the college, it was always 'Daddy's' office the first sought to renew their college experiences." He was great in love and kindness, and had he enjoyed the privilege of a broader early education, he would have been one of the great men of our profession.

